# Epididymis Response Partly Compensates for Spermatozoa Oxidative Defects in snGPx4 and GPx5 Double Mutant Mice

**DOI:** 10.1371/journal.pone.0038565

**Published:** 2012-06-14

**Authors:** Anaïs Noblanc, Manon Peltier, Christelle Damon-Soubeyrand, Nicolas Kerchkove, Eléonore Chabory, Patrick Vernet, Fabrice Saez, Rémi Cadet, Laurent Janny, Hanae Pons-Rejraji, Marcus Conrad, Joël R. Drevet, Ayhan Kocer

**Affiliations:** 1 Genetics Reproduction & Development laboratory, CNRS UMR 6293 - INSERM U1103 - Clermont Université, Aubière, France; 2 Institut Curie, Paris, France; 3 Laboratoire d'Assistance Médicale à la Procréation, Département gynécologie-obstétrique, Hôpital Porte Madeleine, Orléans, France; 4 CHU Estaing, Assistance Médicale à la Procréation, Clermont-Ferrand, France; 5 German Center for Neurodegenerative Diseases, Munich, Germany and Helmholtz Zentrum München, German Research Center for Environmental Health, Institute of Developmental Genetics, Neuherberg, Germany; University of Texas Health Science Center at San Antonio, United States of America

## Abstract

We report here that spermatozoa of mice lacking both the sperm nucleaus glutathione peroxidase 4 (snGPx4) and the epididymal glutathione peroxidase 5 (GPx5) activities display sperm nucleus structural abnormalities including delayed and defective nuclear compaction, nuclear instability and DNA damage. We show that to counteract the GPx activity losses, the epididymis of the double KO animals mounted an antioxydant response resulting in a strong increase in the global H_2_O_2_-scavenger activity especially in the cauda epididymis. Quantitative RT-PCR data show that together with the up-regulation of epididymal scavengers (of the thioredoxin/peroxiredoxin system as well as glutathione-S-transferases) the epididymis of double mutant animals increased the expression of several disulfide isomerases in an attempt to recover normal disulfide-bridging activity. Despite these compensatory mechanisms cauda-stored spermatozoa of double mutant animals show high levels of DNA oxidation, increased fragmentation and greater susceptibility to nuclear decondensation. Nevertheless, the enzymatic epididymal salvage response is sufficient to maintain full fertility of double KO males whatever their age, crossed with young WT female mice.

## Introduction

In mammalian reproduction, either naturally or by assisted reproductive technologies (ART), gamete quality is a key criterion. Sperm DNA integrity is a particularly important parameter concerning the contribution of the male gamete to successful reproduction. There are many reports of adverse clinical effects including depressed fertility, an increased incidence of miscarriage, and offspring morbidity following degradation of paternal chromosomes [1]–[13]. To avoid these potentially adverse conditions, eutherian mammals evolved elaborate processes to protect the paternal chromosomes from loss of integrity, the most obvious being the highly compacted state of the sperm nucleus. Sperm nucleus compaction is a process that is mainly achieved during testicular spermiogenesis by the extensive replacement of nuclear histones by smaller basic proteins, the protamines [14], [15]. During the drastic cyto-differentiation step occurring at the end of mammalian spermatogenesis, compaction of the sperm haploid genome to approximately one tenth the size of the nucleus of any somatic cell serves two major purposes. It provides optimal velocity of spermatozoa and protects the paternal chromosomes from mutagenic effects of both intrinsic and extrinsic origins. At the end of the testicular spermatogenetic program, sperm nucleus compaction is not complete, but proceeds further as spermatozoa pass through the epididymis tubule. During this post-testicular step of sperm maturation, increased nucleus compaction is achieved by osmotic regulation/water resorption and intense disulfide bridging of thiol-containing protamines [16]–[23].

The recent generation of knockout mouse models has allowed a better understanding of how disulfide-bridging events drive this aspect of post-testicular sperm nucleus enhanced compaction. Bi-functional glutathione peroxidase enzymes that can work either as H_2_0_2_/LOOH-recycling enzymes or disulfide isomerases were shown to play key roles in this process. On the one hand, mice lacking the sperm nucleus-located isoform of glutathione peroxidase 4 (snGPx4, a selenium-dependent GPx) present a delay in post-testicular (i.e., proximal epididymis) sperm nuclear compaction [24]. This phenotype demonstrated snGPx4 to be an active disulfide isomerase whose task is to increase sperm nucleus compaction through H_2_O_2_-mediated (or/and LOOH-mediated) oxidation of protamines during early spermatozoa epididymal journey [24], [25]. On the other hand, we have shown previously that deletion of the epididymal luminal H_2_O_2_-scavenger (GPx5, a selenium-independent GPx) resulted in a transient increase in caput epididymal sperm DNA compaction [26]. This effect probably resulted from increased protamine disulfide-bridging *via* snGPx4 because of the luminal increase in H_2_O_2_ and/or LOOH availability due to the lack of GPx5 activity [26], [27]. Thus, we have proposed that GPx5 *via* its epididymal luminal scavenger function acts as a coordinator enzyme that controls the concentration of H_2_O_2_/LOOH in the caput lumen, thereby defining the optimal disulfide-bridging activity of snGPx4 in the caput territory. In addition, throughout the epididymis and especially in the cauda territory, the ROS-scavenging function of GPx5 protects epididymal transiting and cauda-stored spermatozoa form oxidative injuries [26], [27]. To analyze further the importance of these two enzymes in the physiology of the epididymis and in sperm maturation, we have generated transgenic mice lacking both snGPx4 and GPx5. We present here the epididymal and spermatozoa phenotypes of these double mutant animals.

## Results

### Generation of Double Transgenic sngpx4;gpx5^−/−^ Mice

**Figure 1:**
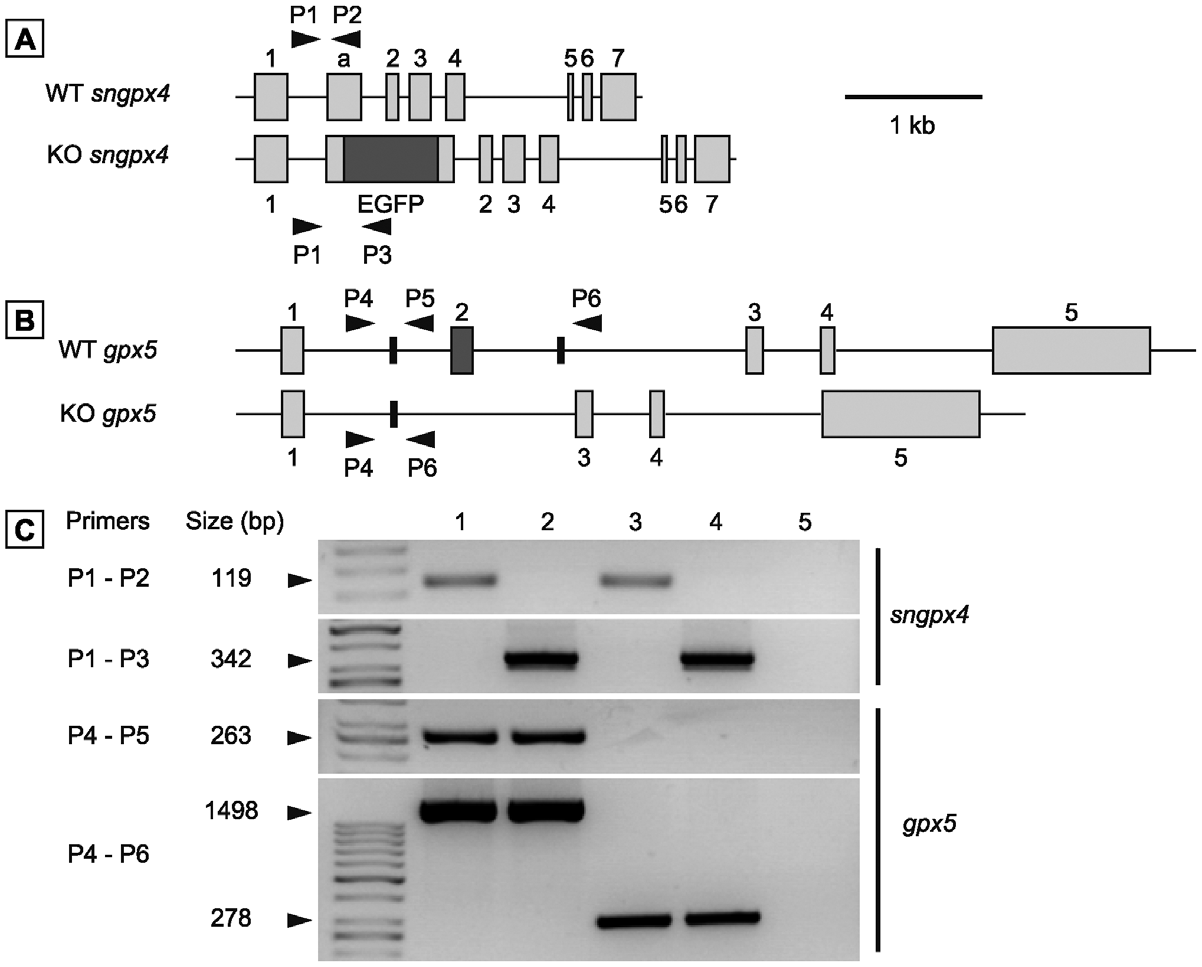
Generation of *sngpx4;gpx5*-deficient mice. A and B: Diagramatic representations of mouse *GPx4* (A) and *GPx5* (B) gene organization (upper diagrams in A and B) and of their engineered version found in the single KO animals (lower schemes in A and B) (1, 20). Transgenic *sngpx4^−/−^* animals bear an EGFP reporter cassette (20), while transgenic *gpx5^−/−^* animals carry a deleted exon 2 (1). Grey boxes (1,a and 2 to 7 for *sngpx4* and 1 to 5 for *gpx5*) indicate the 8 or 5 coding exons, respectively. Bold arrowheads indicate the relative positions of the various primers used thereafter in genotyping PCR amplifications. C: Typical PCR experiments carried out on genomic DNA extracted from animal fingers used to select homozygous *sngpx4;gpx5^−/−^* (DKO) animals. Size (in bp) of the expected PCR products for each primer pair used is given in the left margin. The two upper panels show *sngpx4* amplifications, while the two lower panels show *gpx5* amplifications. Lane 4 (DKO) stands for double knock-out animals, while lane 5 is a negative control in which no genomic DNA was added to the reactions. Primer sequences are given in Table 2. Lanes 1, 2 and 3 are positive control amplifications carried out with genomic DNA from wild type, homozygous *sngpx4^−/−^* and homozygous *gpx5^−/−^* animals, respectively.

Null mice for snGPx4 and GPx5 were generated following crosses of inbred *sngpx4^−/−^* animals [24] and *gpx5^−/−^* animals [26] that were initially produced in the same C57bl/6 genetic background. Genotyping data shown in Figure 1 illustrate that the homozygous derived *sngpx4;gpx5^−/−^* animals carry the transgenic constructs and, therefore, are devoid of functional snGPx4 and GPx5 genes. As for each single KO, simultaneous *sngpx4* and *gpx5* inactivation had no impact on the organization of the epididymis tissue (as evidenced by histological observation) or caput or cauda sperm counts (monitored at 4 months of age), suggesting that lack of snGPx4 and GPx5 expression does not affect sperm production and epididymal transit and function (not shown).

### Spermatozoa of Double Mutant Male Mice Show Impaired Sperm DNA Compaction

**Figure 2:**
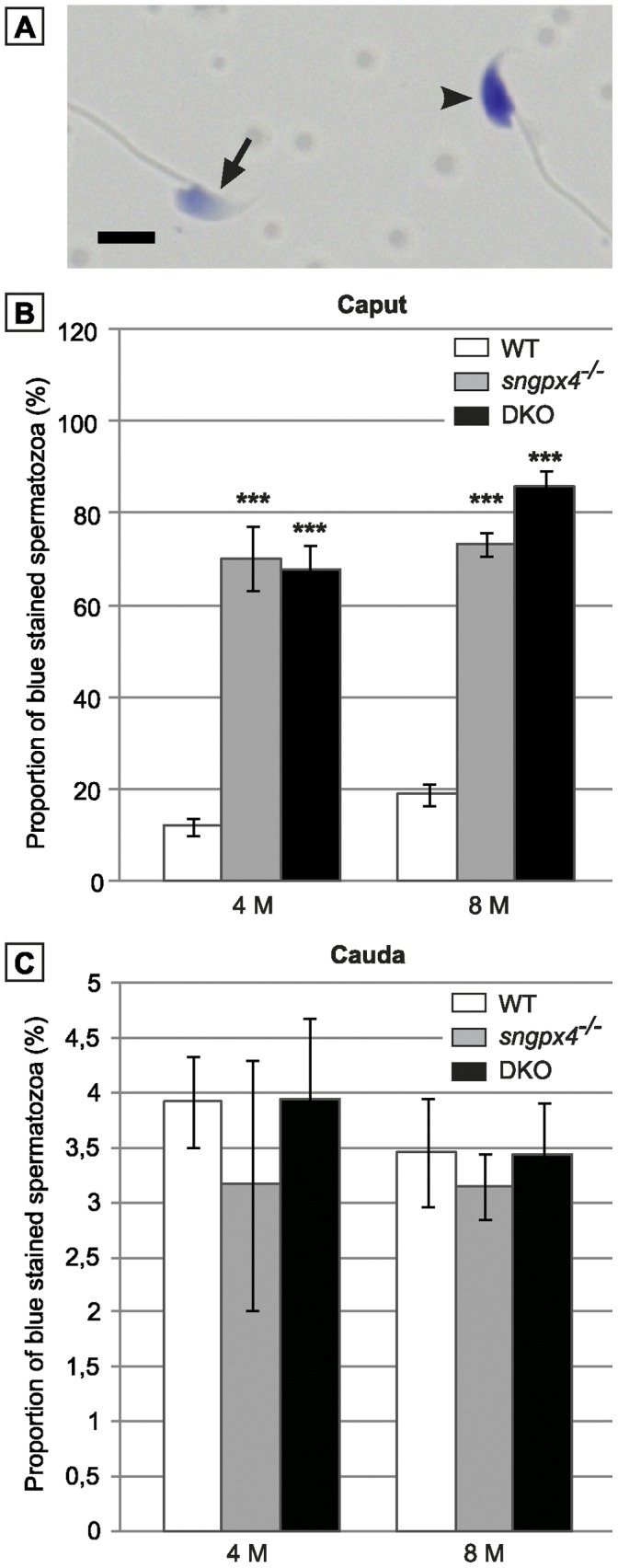
Evaluation of sperm DNA condensation using toluidine blue staining. A: The photograph shows a typical result of toluidine blue staining on a decondensed caput sperm DNA (arrowhead) compared to a condensed caput sperm DNA (arrow). Scale bar = 5 µm. B and C: Histograms plotting the proportion of decondensed sperm nucleus found in WT, *sngpx4^−/−^* and DKO caput sperm population (B) and cauda sperm population (C) in animals aged 4 or 8 months. (Mean +/− SEM; n = 5; ***: p≤0.001 compared to WT).

As shown by toluidine blue staining in caput and cauda sperm of the double KO animals at 4 months and 8 months of age, a large proportion (up to 80%) of the double mutant caput sperm population showed defective nuclear condensation (Figure 2A and 2B). However, sperm DNA compaction resumed an apparently normal level in cauda-collected spermatozoa of the double mutants (Fig. 2C).

### Impaired Sperm DNA Compaction in the Double Mutant Animals is Associated with a Giant Head Sperm Phenotype

**Figure 3:**
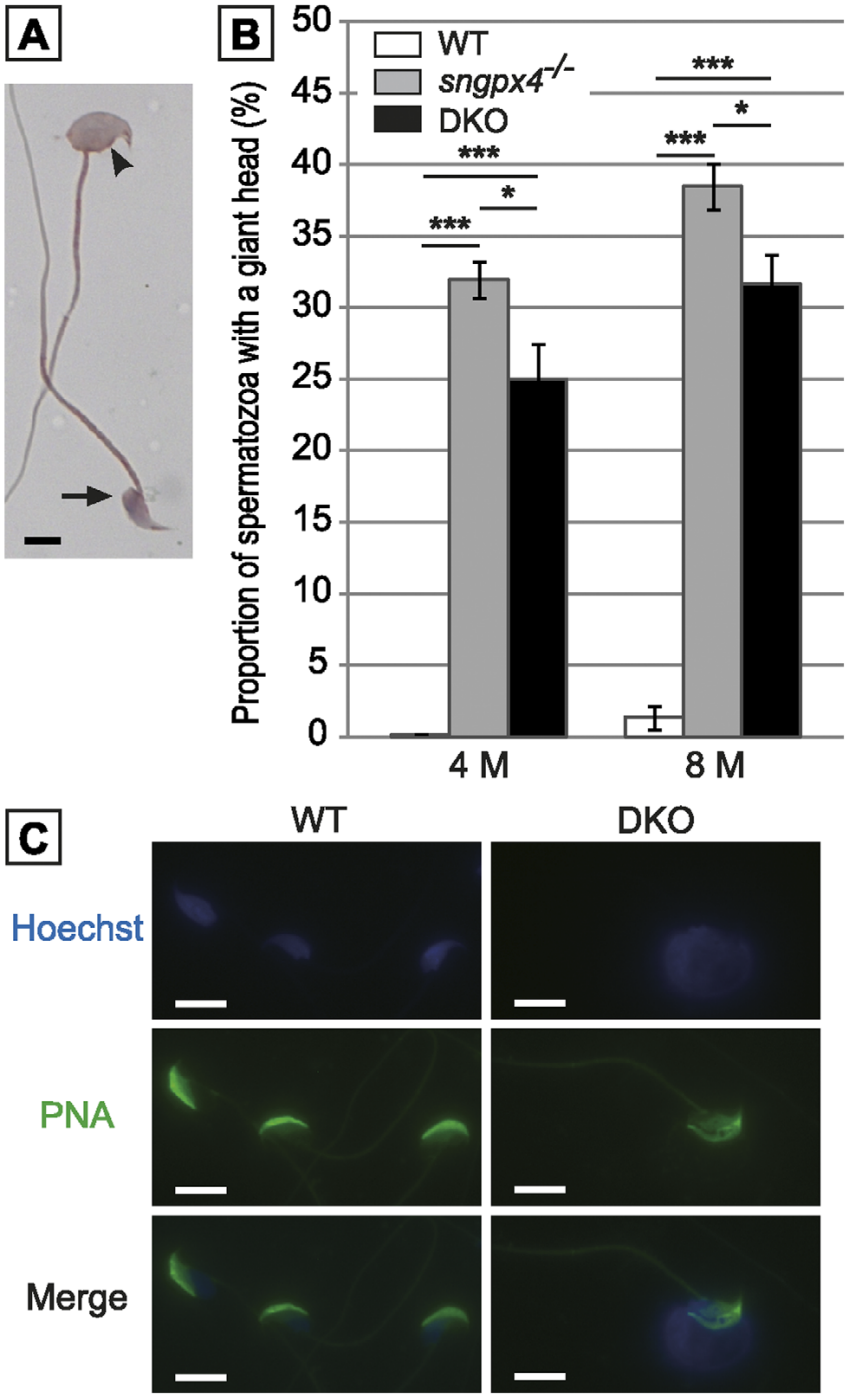
Morphological evaluation of caput sperm heads using Schorr staining. A: Photograph shows sperm head morphology with (arrowhead) or without (arrow) alterations found in caput epididymidis. Scale bar = 5 µm. B: Histograms plotting the proportion of caput sperm showing a giant head phenotype in WT, *sngpx4^−/−^* and DKO animals aged 4 or 8 months. (Mean +/− SEM; n = 5; ***: p≤0.001 compared to WT) C: Micrographs show WT (left panels) and DKO (right panels) caput spermatozoa stained either with Hoechst to localize the nucleus compartment (upper panels) or with PNA-Alexa488 to visualize the acrosome compartment (median panels). PNA-Alexa488 and Hoechst stains are merged on the lower panels. Scale bar = 5 µm.

Shorr staining (Figure 3A) showed that 30 to 35% of the caput sperm population had a giant head phenotype irrespectively of mice age (4 or 8 months, Figure 3B). Again, this is a caput restricted sperm phenotype, because less than 0.2% of the cauda sperm population exhibited this morphological alteration (data not shown). Using PNA-Alexa and Hoechst staining to detect the acrosomal and the nuclear sperm compartments, respectively, we show in Figure 3C, that the giant sperm head phenotype of the double mutant mice was essentially due to nuclear expansion (as evidenced by the Hoechst staining) while the acrosome-staining was not dramatically different from that of the WT spermatozoa.

### Nuclear Susceptibility to Reducing Conditions of Double Mutant Cauda Spermatozoa

**Figure 4:**
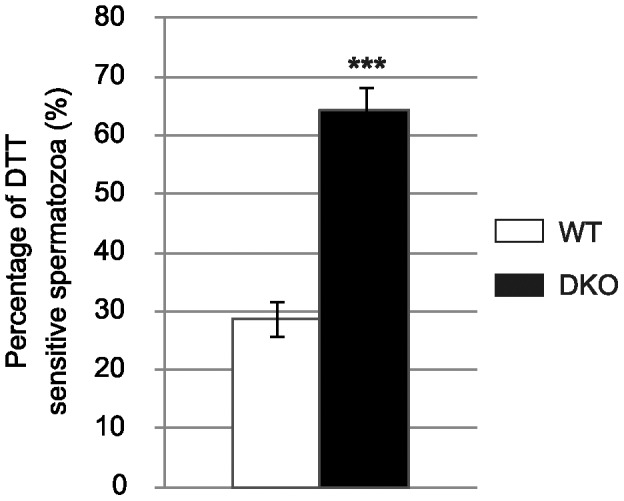
DKO cauda spermatozoa fragility. Histograms showing the proportion of WT and DKO cauda spermatozoa with a giant head phenotype after treatment of the sperm preparations with 2 mM DTT. (Mean +/− SEM; n = 5; ***: p≤0.001 compared to WT).

Although sperm nucleus size was similar to that of WT in the cauda compartment of double mutant animals (see Figure 2C), Figure 4 shows that these spermatozoa remained very sensitive to reducing conditions such as treatment with dithiotreitol (DTT). When applied on cauda-retrieved double mutant spermatozoa, it provoked nuclear expansion and the reappearance of the giant head phenotype for a larger proportion of spermatozoa compared to WT animals. This data reveals that cauda sperm nuclei of double mutant animals did not reach their optimal and solid condensed state compared to WT animals.

### Nuclear Expansion in Spermatozoa of Double Mutant Animals is neither Due to Defective Protamination nor to Decreased Disulfide Bridging

**Figure 5:**
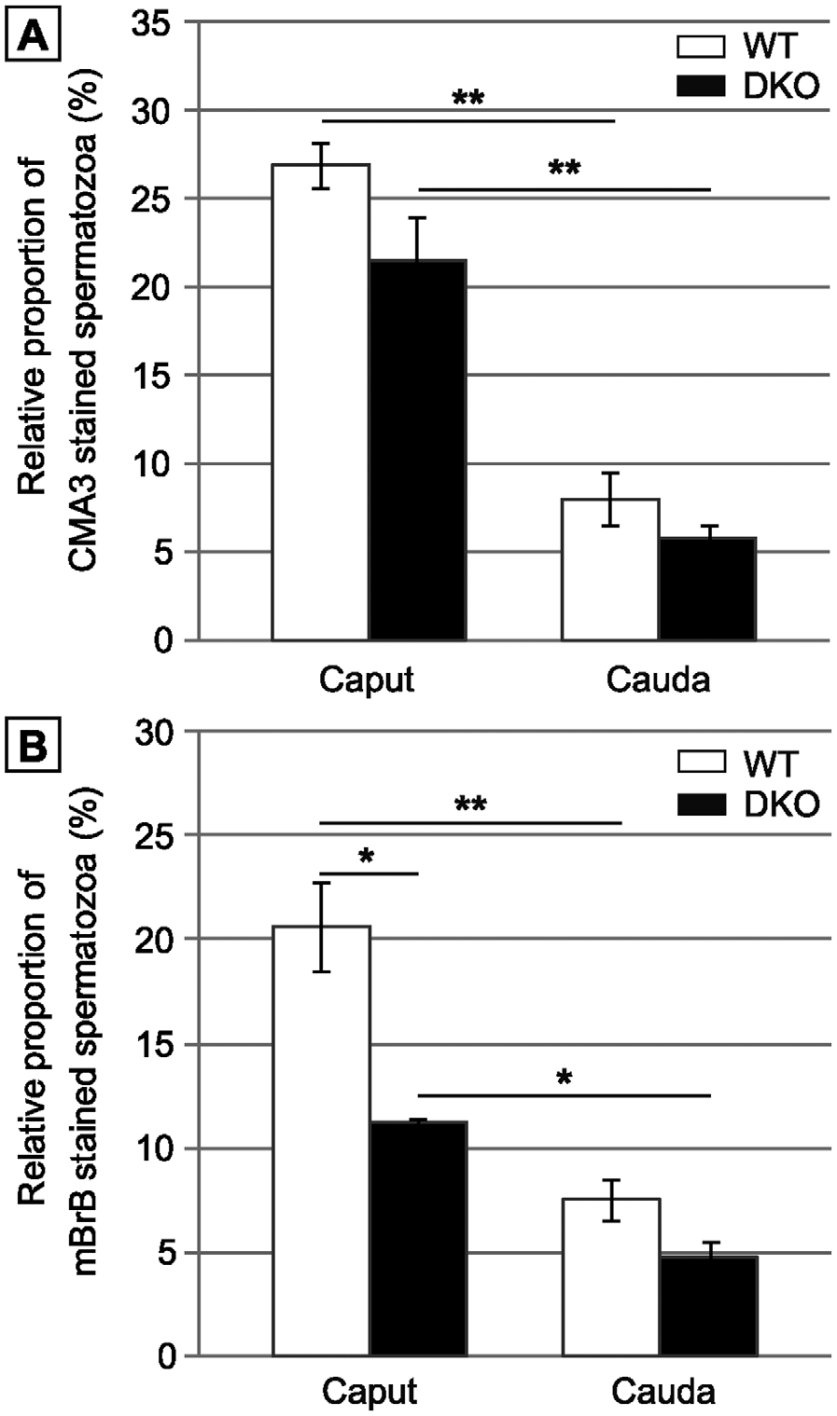
Evaluation of spermatozoa integrity by flow cytometry. A: Protamine association with sperm chromatin determined by chromomycin A3 (CMA3) staining. Histograms show the proportion of CMA3 incorporated in caput and cauda sperm of WT versus DKO animals aged 4 months. B: Disulfide bonds/free thiol quantification by monobromobimane (mBrB) staining. Histograms showing the incorporation of mBrB in caput and cauda sperm of WT versus DKO animals aged 4 months. (Mean +/− SEM; n = 6; *: p≤0.05; **: p≤0.01).

Since we recorded no differences in the spermatozoa phenotype of the double mutant animals at 4 and 8 months, all further experiments were carried out on spermatozoa from animals aged 4 months. Because snGPx4 is located in the sperm nucleus during spermiogenesis, it may be argued that its absence could somehow impair the process of histone replacement by protamines, eventually leading to defective sperm nuclear protamination in the double mutant. We used flow cytometry and chromomycin A3 as a probe to evaluate indirectly the protamine content of WT and double mutant spermatozoa. Figure 5A first shows that sperm nuclear compaction was increased during spermatozoa epididymal journey from the caput to the cauda irrespective of the genetic background. This Figure also indicates that there was no significant difference in the percentage of recorded fluorescence due to chromomycin A3 staining both in caput and cauda double mutant spermatozoa when compared to WT spermatozoa. Defective testicular protamination is therefore unlikely to explain sperm nuclear decondensation in the double mutants.

To obtain an evaluation of epididymal disulfide-bridging activity, flow cytometry and a monobromobimane probe were used to monitor the free thiol content of sperm proteins in WT and double mutant animals. Figure 5B first illustrates increase in sperm disulfide-bridging events during spermatozoa epididymal journey from the caput to the cauda irrespective of the genetic background. In addition, and in contrast to what might have been logically expected in a context of defective snGPx4 disulfide bridging activity, Figure 5B also shows that caput and, to a lesser extent cauda sperm samples of double mutant animals, contained less free thiol groups than WT controls. Since monobromobimane staining is inversely correlated with the number of disulfides, these data suggest that increased disulfide-bridging events took place in the epididymis (particularly in the caput compartment) of double mutant animals despite the absence of the snGPx4 player. This suggests that the epididymis of the double mutant animal has somehow found a way to enhance its global disulfide isomerase activity.

### The Epididymis of Double Mutants Compensates for the Lack of ROS-scavenging Activity

**Figure 6:**
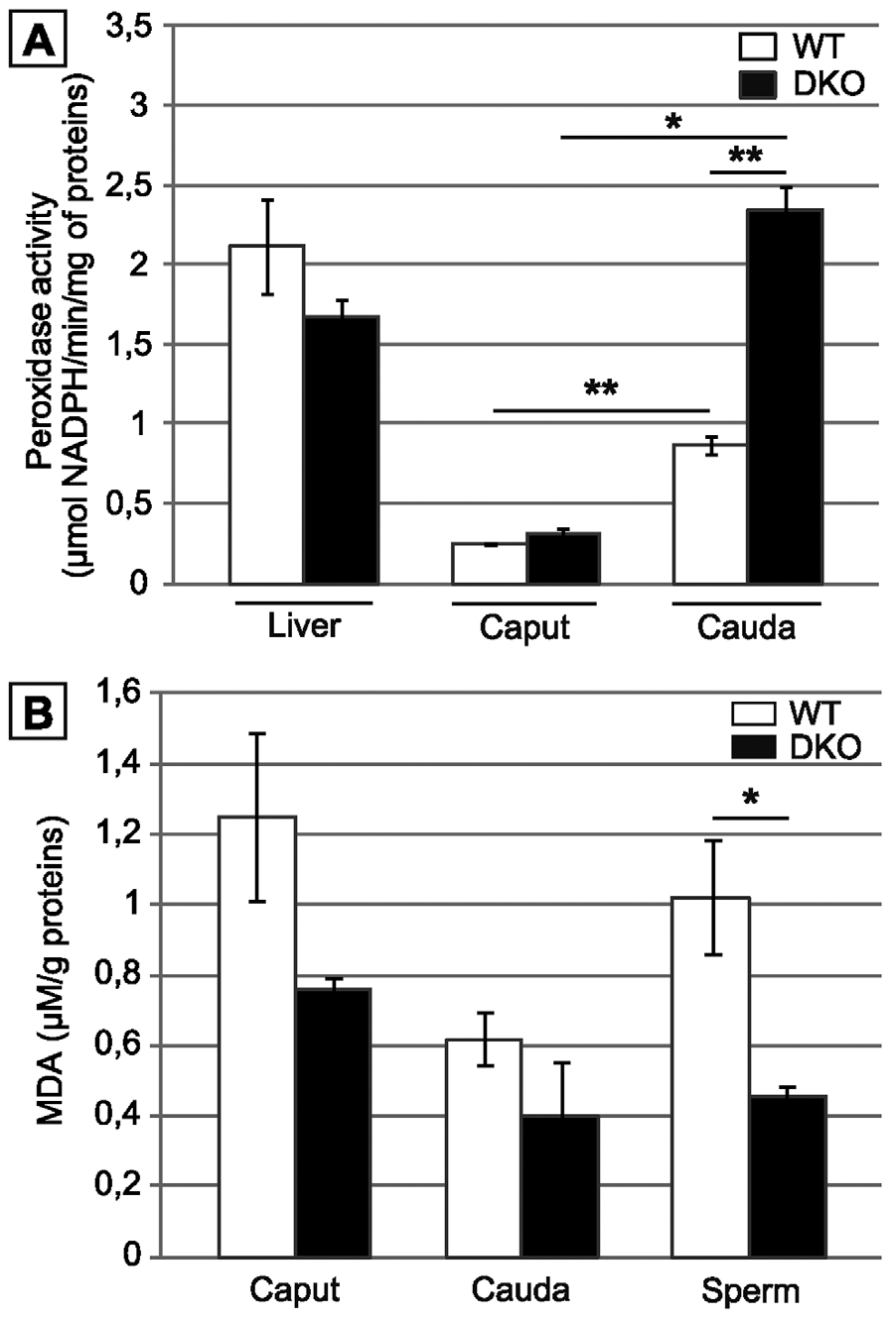
snGPx4;GPx5 deficiency leads to a strong epididymis anti-oxidant response. A: Histograms show global H_2_O_2_-scavenging activity using H_2_O_2_ as a substrate in caput or cauda epididymis tissue extracts from WT or DKO animals aged 4 months. To evaluate the extent of the epididymis anti-oxidant response global H_2_O_2_-scavenging activity in liver extracts of the same animals are shown on the left. (Mean +/− SEM; n = 5; **: p≤0.01 compared to WT) B: Malondialdehyde (MDA) measurements on caput and cauda epididymal tissues as well as on cauda-collected spermatozoa from WT and DKO animals aged 4 months. (Mean +/− SEM; n = 3; *: p≤0.05 compared to WT).

Figure 6A shows that in the double mutant animals, global H_2_O_2_-scavenging activity was up-regulated especially in the cauda compartment. The global H_2_O_2_-scavenging activity recorded in the double mutant cauda territory exceeded by far that recorded in the same compartment in WT animals. As a physiological indicator, Figure 6A, also shows that the global H_2_O_2_-scavenging activity recorded in the epididymis of double mutant animals was quite comparable to that found in liver, a tissue well-known for its high ROS-recycling metabolism. It is also important to note that, in the double mutant animals, the epididymis but not the liver was engaged in this global antioxidant response. As a result, the content in malonyldialdehyde (MDA), an end-point marker of lipid peroxidation, was significantly lower in spermatozoa of double mutant animals when compared to WT (Figure 6B). In caput and cauda tissue extracts, the MDA content was similar when WT samples were compared to KO samples. This suggests that the surnumerary protection against lipid peroxidation engaged by the cauda epididymis was of a secreted nature or already present in the vicinity or on the sperm cell itself.

### Both H_2_O_2_ Scavengers and Disufilde Isomerases are Up-regulated in the Epididymis of the Double Mutant Animals

To determine which ROS-recycling enzyme(s) and disulfide-bridging enzymes(s) were up-regulated in the epididymis of the double mutant animals, we used qRT-PCR to monitor the expression of various genes encoding these classes of proteins (see Table 1) in the caput and cauda epididymidis compartments of WT and double mutant male mice. Concerning ROS-recycling enzymes (ROS-scavengers) we found that overexpression of the classical cauda epithelial GPxs (ie. GPx1, GPx3 and the cellular isoform of GPx4 [cGPx4]) as well as catalase, all being cytosolic enzymes, were not responsible for the extra scavenging capacity of this territory in the double mutant animals. Surprisingly, we even recorded a decrease in the expression of these classical H_2_O_2_- or/and LOOH-recycling GPx genes both in the caput and cauda territories of the double mutant animals compared to WT. The extra H_2_O_2_ (or LOOH) recycling/consuming activities switched on in the epididymis of the double mutant animals were therefore not of the classical GPx/Catalase types. Rather, glutathione S transferases (GSTµ and GSTπ), a peroxiredoxin (PRDX3) and thioredoxin/thioredoxin-like genes such as TXN1 and TXNL1 were found up-regulated in the epididymis of the double mutant animals. We recorded some regional differences in the overexpression of these ROS-recycling activities since TXNL1 overexpression appeared to be a caput-restricted response while TXN1 and PRDX3 overexpression were recorded solely in the cauda territory. Regarding the response of the epididymis in terms of disulfide-bridging activities, we monitored the expression of several classical disulfide isomerases and we show in Table 1 that PDIA3 (ERP57), PDIA5, PDIA6 (TXNDC7), PDIA10 (ERP44/TXNDC4) and PDIA11 (TXNDC1) were up-regulated in caput and/or cauda tissue samples of the double mutant animals compared to WT.

**Table 1:** Evaluation of the expression of various ROS-scavenger and disulfide isomerase genes using real-time PCR on tissue extracts of caput and cauda epididymidis from WT and DKO animals aged 4 months.

	Caput		Cauda	
Gene	Significance	Fold	Significance	Fold
*gpx1*	*	**−**1,52		=
*gpx3*		=		=
*gpx4*	**	**−**1,84	p = 0,0635	−2,07
*catalase*	*	**−**1,35		=
*sod3*		=	*	−3,36
*prdx1*		=		=
*prdx2*		=		=
*prdx3*		=	**	+1,79
*prdx4*		=		=
*prdx5*		=		=
*prdx6*		=		=
*txn1*		=	*	+1,47
*txn2*		=		=
*txnl1*	**	**+**1,26		=
*txndc2* (*sptrx1*)		=		=
*txndc3* (*sptrx2*)		=		=
*txndc8* (*sptrx3*)		=		=
*gst µ*	*	**+**1,34	p = 0,0556	+1,37
*gst π*	*	**+**1,38	p = 0,0556	+1,36
*pdia3 (erp57)*		=	*	+1,56
*pdia4* (*erp72*)		=		=
*pdia5*	p = 0,0556	+1,28	*	+1,44
*pdia6* (*txndc7*)	**	**+**1,36	p = 0,0556	+1,15
*pdia10* (*txndc4*)	**	**+**1,31	**	+1,3
*pdia11* (*txndc1*)		=	*	+1,22

For each gene, the change in its expression in DKO *versus* WT male mice is indicated by + or − symbols, followed by the fold between the WT and DKO gene expressions ( = : not significantly different; +: increased; −: decreased; n = 5; *: p≤0.05; **: p≤0.01).

### Spermatozoa of Double Mutant Animals Show Increased DNA Fragmentation associated with DNA Oxidative Damage

**Figure 7:**
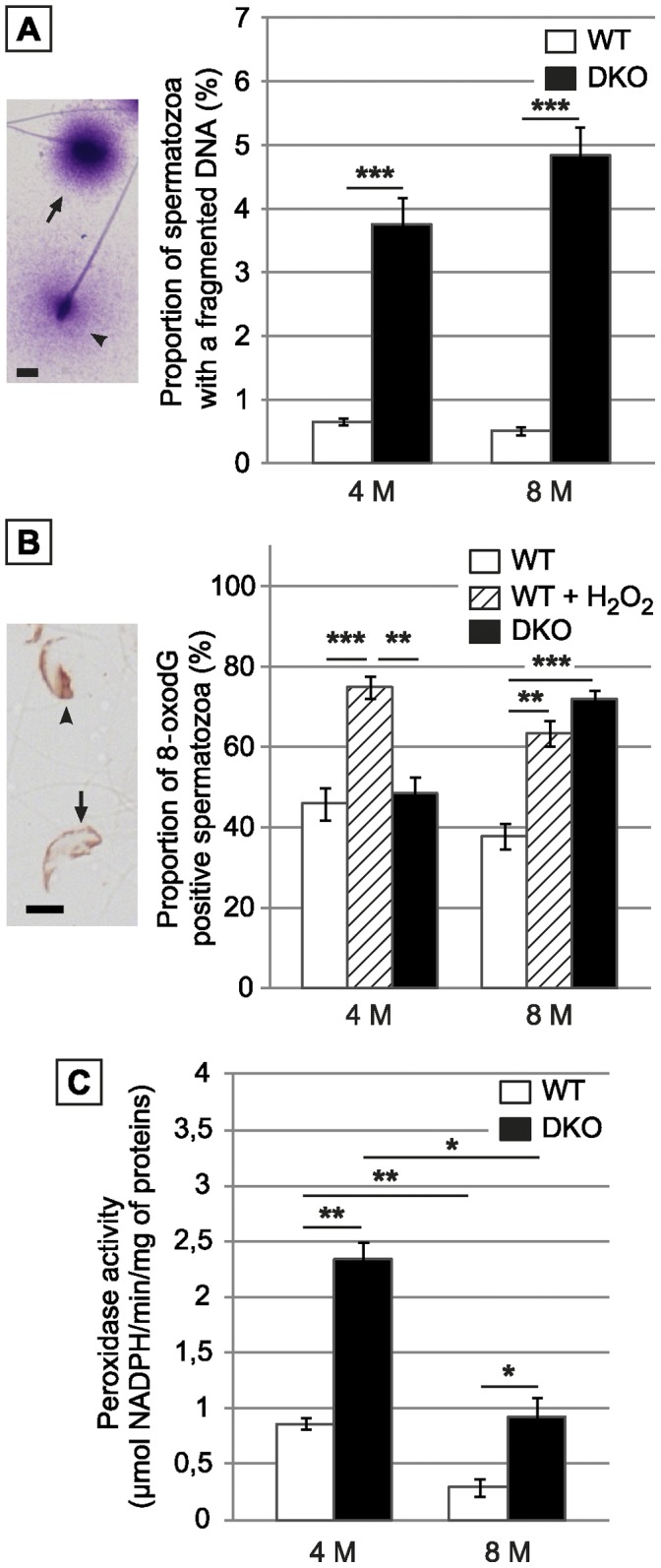
Cauda-retrieved spermatozoa of DKO animals suffer oxidative damage. A: Left panel: Typical picture of fragmented (arrowhead) or non-fragmented (arrow) sperm nucleus as shown by the modified Sperm Chromatin Dispersion Assay. Scale bar = 5 µm. Right panel: Histograms show the proportion of WT and DKO cauda collected spermatozoa with a fragmented DNA in animals aged 4 or 8 months. B: Left panel: Typical immunodetection of the nuclear adduct 8-oxodG in cauda epididymidis-retrieved spermatozoa preparations from DKO male mice aged 8 months. Scale bar = 5 µm. Right panel: Histograms show the percentage of 8-oxodG positive spermatozoa in cauda epididymidis-retrieved spermatozoa preparations, respectively from WT, positive control (WT spermatozoa treated with H_2_O_2_) and DKO male mice aged 4 and 8 months. (Mean+/− SEM; n = 5; *: p≤0.05; **: p≤0.01; ***: p≤0.001). C: Histograms show global H_2_O_2_-scavenging activity using H_2_O_2_ as a substrate in cauda epididymidis tissue extracts from WT or DKO animals aged 4 or 8 months. (Mean +/− SEM; n = 5; *: p≤0.05; **: p≤0.01).

Based on a modified method of the classical sperm chromatin dispersion assay (SCDA, [28]) Figure 7A illustrates that cauda spermatozoa of double mutant animals presented a higher level of DNA fragmentation than WT spermatozoa at the same ages. Figure 7B also shows that for double mutant animals aged 4 months the level of sperm DNA oxidative damage was well controled by the epididymis antioxidant response since cauda spermatozoa were as reactive to an 8 oxo-dG antibody as WT spermatozoa. However, the situation was rather different with spermatozoa collected from older double mutant animals (8 months) where a high proportion of spermatozoa revealed oxidized guanine residues typical of DNA oxidative alterations. Figure 7C shows that this weaker protection of cauda collected spermatozoa in older animals was probably due to a general physiological decrease in the cauda epididymidis antioxidant response since we show that at this age (8 months), global H_2_O_2/_LOOH-recycling activity was reduced by 2.5-fold both in WT and double mutant cauda epididymidis protein extracts compared to the situation in animals aged 4 months.

### The Epididymis Antioxidant Response in the Double Mutant is Sufficient to Preserve Fertility

**Figure 8:**
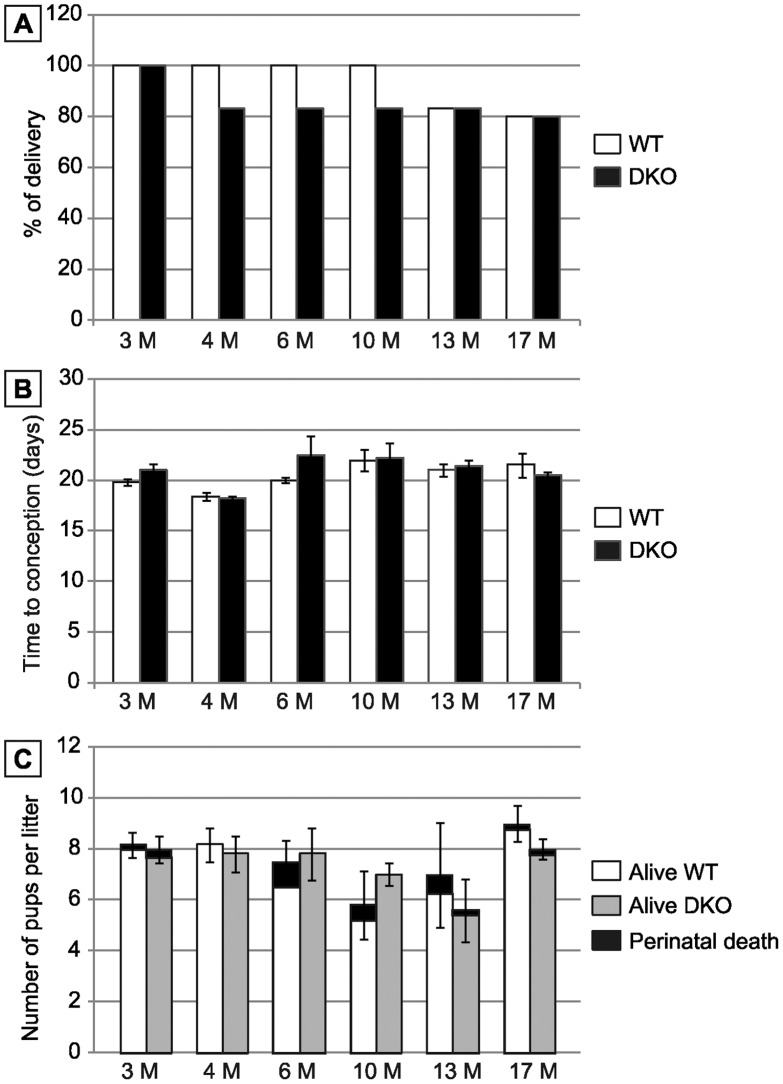
Impact of snGP4 and GPx5 deletions on mouse fertility. A: Histograms plotting the percentage of delivery of several matings between WT or DKO males and WT females (aged 3 months) according to male age given in months. B: Histograms plotting the delay to conception recorded in matings carried out with WT female mice (aged 3 months) and WT or DKO male mice aged 3 to 17 months. (Mean +/− SEM; n = 6). C: Histograms plotting the number of pups per litter for WT or DKO males (open bars and grey bars, respectively) according to male age given in months. Black sections indicate the number of dead pups (perinatal mortality). (Mean +/− SEM; n = 6).

To investigate the effect of the absence of snGPx4 and epididymal GPx5 expression on male fertility, mice of both genotypes (*wt* and *sngpx4;gpx5−/−*) were mated with *wt* female C57Bl/6 mice. Mating was conducted with animals at optimal reproductive age (3 months). No changes in mating behaviour were noticed. Three-month old double mutant male mice were found fertile with litter sizes (pups 8.00±0.52, n = 6) comparable to those of WT mice at the same age (pups 8.17±0.48, n = 6) (Figure 8A). More crosses were carried out with older male mice and *wt* young females to determine if the absence of both GPx had a greater effect after aging. Figure 8A shows that in males up to 17 months of age there were no statistically significant differences in litter sizes between *sngpx4;gpx5*−/− and WT backgrounds. Time to gestation was also monitored and found to be comparable to that of WT crosses (Fig. 8B). Number of dead pups and perinatal mortality were monitored for each cross and found to be statistically comparable in WT and double mutant animals (Fig. 8C).

## Discussion

We and other authors, have already proposed that sperm nuclear compaction is enhanced during epididymal descent by the combined actions of the snGPx4 disulfide isomerase and the luminal epididymal GPx5 scavenger that control H_2_O_2_ (or LOOH) availability, therefore determining the level of disulfide-bridging events on thiol-containing sperm protamines [24]–[27]. We report here on the generation of a mouse strain in which both GPxs (snGPx4 and GPx5) were invalidated. In agreement with the tissue- and cell-restricted expression of both genes, and similarly to each single knock-out, lack of snGPx4 and GPx5 expression has no impact on animal viability. Using light microscopy, we recorded no difference in the organization and cytology of epididymal and testicular tissues of the double mutant animals when compared to WT animals (not illustrated). In addition, sperm cell number and sperm cell viability were found to be identical to those of control animals regardless of age. Since we expected from the respective single knock-outs [25], [26] that spermatozoa from the double mutant animals would present nuclear structural defects, we focused our investigation on caput and cauda spermatozoa of the double mutants. We first show that already at 4 months of age and as early as in the caput territory about 80% of double mutant spermatozoa are concerned by nuclear decompaction. Compared to the single *gpx5^−/−^* model, the situation in the double mutant is rather different since caput sperm of *gpx5^−/−^* animals showed increased nuclear compaction attributed to a higher availability of H_2_O_2_/LOOH for the disulfide bridging activity of the snGPx4 enzyme [26]. The situation is however identical to that of caput spermatozoa of the single *sngpx4^−/−^* strain at the same age as reported earlier by Conrad et al (2005) [25]. These data suggest that in the caput territory of the double mutant animals, lack of GPx5 activity does not worsen the state of sperm nuclear compaction observed in the single *sngpx4^−/−^* model [24]. Alternatively, it may also indicate that defective GPx5 ROS-scavenging function has been compensated by other means in the double mutant animals. This is supported by our observations that several enzymatic scavengers are up-regulated both in the caput and the cauda epididymis of the double mutant animals compared to WT controls.

The nuclear decompaction we observed in caput-collected sperm of the double mutants was found to be transient since an apparently normal WT-like nuclear compaction was seen in cauda sperm, a situation also reported earlier for the snGPx4 KO [24], [29]. We show here that low caput sperm nuclear compaction was associated with a giant-head phenotype affecting one third of the caput sperm population. We also show that this giant-head sperm phenotype is correlated with nuclear decondensation. Sperm nuclear instability in the double mutant was not due to defective protamination or to decreased disulfide-bridging. On the contrary, for the latter, despite the absence of the snGPx4 disulfide isomerase activity, total sperm disulfide content was significantly greater in double mutants than in control WT animals, especially in the caput compartment. This observation again supports the idea that other activities, probably disulfide isomerases, have been switched-on to compensate for the absence of snGPx4 during spermatozoa epididymal transit in the double mutant animals. In agreement with this hypothesis we found that several protein disulfide isomerases or PDI-related proteins were up-regulated both in the caput and in the cauda compartments of the double mutant animals. Namely, PDIA3 (ERP57), PDIA5, PDIA6 (TXNDC7), PDIA10 (TXNDC4/ERP44) a PDI-chaperone protein which was shown to be involved in the control of oxidative protein folding [30] and PDIA11 (TCXNDC1), were found significantly up-regulated in the epididymis of the double mutant animals. These protein disulfide isomerases are already known to be expressed at significant levels in the mouse epididymis [31]. In particular, PDIA5 over-expression in the epididymis of the double mutant animals could be a logical response of the tissue to the absence of the caput active snGPx4 because PDIA5 was reported to be preferentially expressed in the distal caput epididymis [31]. Although the increase in protein disulfide isomerase expression allowed recovery of a normal nuclear compaction state in cauda-stored spermatozoa of double mutant animals we show here that the nucleus of these spermatozoa still remained fragile when challenged by a reducing agent such as DTT. DTT treatment of spermatozoa provoked the reappearance of the giant-head phenotype in two-third of the cauda sperm population in 4 month-old double mutant animals, while only 25% of WT cauda-stored spermatozoa were reactive to this treatment. As in the caput compartment of double mutants, the giant-head phenotype of cauda spermatozoa was shown to be associated with nuclear decondensation. These data are in agreement with a very recent report on *sngpx4^−/−^* sperm nuclei which were also found to be susceptible to provoked decondensation [29]. Therefore, despite higher disulfide-bridging events due to the increase in protein disulfide isomerase expression along the epididymal tubule of the double mutant animals, sperm nuclei did not reach its WT-type-like optimal condensed state. This may be related to the inability of these induced disulfide isomerases to work in the sperm nucleus. In addition, it is also possible that cauda sperm DNA instability in the double mutant results from an increase in ROS-mediated DNA alterations (from DNA oxidation to DNA breaks) due to the pro-oxidant environment (i.e., because of the lack of GPx5). This would be comparable to the cauda sperm phenotype reported earlier in the single GPx5 KO [26]. Our data reinforces the idea that an optimal epididymal disulfide cross-linking activity is important to “shield” sperm nucleus against ROS-induced DNA damage. The duality of the situation is that the epididymis uses H_2_0_2_/LOOH to promote sperm-nucleus GPx4 disulfide isomerase activity even though spermatozoa are particularly sensitive to ROS. One explanation may reside in the fact that only a permeant agent such as H_2_O_2_ has the ability to reach the already quite condensed sperm nucleus compartment.

Concerning the antioxidant response of the epididymis in the double mutant, we show here that there was a large increase in H_2_O_2_-scavenging activity especially in the cauda epididymis of the double mutant. Total H_2_O_2_-recycling capacity in the cauda epididymis of the double KO animals was such that it reached and even exceeded the H_2_O_2_-scavenging activity recorded in liver, a tissue that has a considerable H_2_O_2_-recycling coverage because of its high detoxification metabolism. In comparison, global GPx-like activity in cauda epididymidis extracts of the single GPx5 KO animals was reported earlier to be identical to that of the WT controls [26]. Interestingly, in the earlier model, the maintenance of normal H_2_O_2_-scavenging activity in the cauda epididymis was shown to be due to the transcriptional up-regulation of the epithelial cytosolic GPxs (GPx1, GPx3 and cGPx4) as well as catalase [26]. We show here that the adaptive response of the epididymis in double mutant animals was different, because neither the cytosolic epithelial epididymal GPxs nor catalase were found to be transcriptionally up-regulated. On the contrary, compared to control animals, accumulations of epididymal GPx transcripts and to a lesser extend catalase transcripts were significantly lower in the caput and cauda compartments of the double mutants. This behavior may be comparable with recent reports which have shown that prolonged/chronic ROS exposure contributes to the down-regulation of GPx or catalase expression (for example see [32]). SOD3 expression was also found to be significantly reduced in the cauda epididymis territory of the double mutant animals. Since extracellular SOD activity contributes to the generation of luminal H_2_O_2_, it is possible that down-regulation of SOD3 expression is another method by which the epididymis of the double mutant animals regulates accumulation of this ROS around stored spermatozoa. These observations suggest that H_2_O_2_-recycling in the epididymis of the double mutant animals is ensured by other enzymes. In agreement with this hypothesis, we found that several scavengers were transcriptionally up-regulated in the caput and cauda epididymidis compartments of the double mutant, including thioredoxins (TRX)/thioredoxin-like proteins such as TXN1 and TXNL1 as well as a peroxiredoxin (PRX), PRDX3. The thioredoxin/peroxiredoxin (TRX/PRX) couple is the prevalent redox system occurring in a wide variety of tissues and cells [33], [34]. Some PRX have been shown to be more efficient in neutralizing H_2_O_2_ than catalase and the glutathione/glutathione peroxidase (GSH/GPx) system [35], [36]. It has also been reported that they are regenerated a lot faster than other redox proteins [37]. PRDX3 is a mitochondrial peroxidase that was shown in a different context, the eye lens epithelia, to be highly and specifically induced by low levels of H_2_O_2_ and as such, is considered as a gene acutely responsive to increased H_2_O_2_ levels [38]. Expression of PRX proteins throughout the mouse epididymis was reported earlier and PRDX3 is, together with PRDX1, PRDX5 and PRDX6, significantly expressed in the caput and cauda compartments [31]. Our analysis shows that only PRDX3, but not the other epididymis-expressed PRDXs, is up-regulated in the cauda territory of double mutant animals. Concerning thioredoxins, TXN1 was also reported to be significantly expressed throughout the epididymis [31], while TXNL1 was found to be weakly expressed in that tissue [31]. Because of the low profile of the glutathione system in the epididymis, it would not be surprising that the TRX/PRX system may play a significant role in eliminating excess H_2_O_2_ in that compartment. The role of thioredoxin as electron donor in the epididymis has already been proposed when we showed earlier, that GPx5 is a selenium-independent GPx of the 2-cyst-type that uses TRX rather than GSH as an electron donor [39]–[41]. Finally, together with thioredoxins and peroxiredoxin 3, we have found that glutathione-S-transferases (GSTπ and GSTµ) are also significantly up-regulated both in the caput and cauda epididymidis compartments of the double mutant animals. This agrees with previous reports showing that GSTs are expressed at significant levels in mammalian epididymis [31], [42]. Induced expression of multiple forms of GST appears to be an evolutionary response of cells to protect against chemical toxicity and oxidative stress. Therefore, induction of these enzymes in the epididymis of double mutants that lack antioxidant protection is not so surprising. It is interesting to note that different subsets of enzymes are up-regulated in the caput and the cauda compartments of double mutant animals compared to WT. The caput overexpresses TXNL1 and GSTs while the cauda overexpresses TXN1, PRDX3 and GSTs. This stronger antioxidant response of the cauda epididymidis of double mutant animals parallels the higher H_2_O_2_-recycling activity we have recorded in that part of the epididymis and indicates the TXN1/PRDX3 couple as the major contributor to this extra H_2_O_2_-recycling activity.

The ability of the epididymis to respond to oxidative stress even when a major luminal player (GPx5) has been invalidated shows the large capacity of this tissue to deal with ROS. It is interesting to note that the antioxidant response of the double mutant cauda epididymidis is sufficient to protect spermatozoa nucleus from oxidative damage in males aged 4 months, whereas it is not so in males aged 8 months. We provided a logical explanation for this observation since it follows the overall loss in cauda epididymidis antioxidant protection on aging as both WT and double mutant animals show an identical decrease in their global cauda H_2_O_2_-recycling capacity between 4 and 8 months of age. These observations indicate that adaptive responses of the epididymis are inefficient in older animals. Rescue provided by the epididymis antioxidant response in the double mutant context is partly sufficient to correct the deficiency in sperm nuclear disulfide bridging activity and sperm protection against oxidation. Our interpretation of the situation is that the epithelium antioxydant response attempts to counteract a H_2_O_2_/LOOH-rich luminal environment in order to protect itself. Spermatozoa do benefit from the epithelium antioxidant response because we have shown that cauda sperm of the double KO animals present a lower level of lipid peroxidation than WT spermatozoa. However, because sperm cells in the epididymal lumen cannot mount a stress response they suffer internal ROS-mediated damage such as DNA oxidation essentially because H_2_O_2_ can freely go into any cell compartment.

Thus, the epididymal rescue does not appear to be sufficient to completely protect paternal DNA from oxidative insults, hence the sensitivity to reducing condition that provokes nuclear decompaction, the higher level of DNA fragmentation and susceptibility to DNA oxidation upon aging found in cauda-stored spermatozoa of double mutant animals. Although it is likely that the oxidative insults of the sperm nucleus we recorded in the double KO animals occurred in the defective epididymal environment, we cannot at this stage rule out the idea that part of these sperm nuclear ROS-mediated damage start during late spermatogenesis when snGPx4 is normally associated with protamines. However, lack of GPx5 expression only affects the antioxydant capacity of the epididymis lumen (26) and snGPx4 was shown to perform its disulfide isomerase job on sperm protamines during their transit through the caput epididymis (24).

Of note is that the epididymis salvage in the double mutant, together with oocyte capacity to repair paternal DNA disorders, are quite sufficient to maintain reproductive success, as double mutant males irrespective of their age were as fertile as WT males when naturally mated with young female mice. This emphasizes the plasticity and the ability of the epididymis to counteract excessive ROS-mediated sperm degradations. Investigations are in progress to determine whether subfertility is encountered in this model when using *in vitro* fertilization (IVF) which bypass female sperm selection processes [43], [44]. Experiments are also conducted to determine whether or not subfertility is observed when using older oocytes which are known to have lost their optimal DNA repair capacity [45].

In conclusion, the epididymis of mice lacking expression of snGPx4 and GPx5 is able to trigger an antioxidant response and to increase disulfide isomerase expression to compensate for the absence of both proteins. Although antioxidant and disulfide-bridging activities are increased, they do not completely protect the cauda-stored spermatozoa and, particularly, its nuclear compartment that remains unstable, probably as a result of increased DNA oxidative insults. Our data emphasize that normal sperm nucleus condensation should not be considered as an absolute indicator of full nuclear integrity. Therefore, although MSOME (Motile Sperm Organellar Morphological Examination [46]) offers a powerful method to select morphologically normal spermatozoa for ICSI procedures, it is important to find new techniques that could be used in routine ART to evaluate sperm nuclear integrity prior to IVF and ICSI. This agrees with reports showing that a high sperm DNA Fragmentation Index (DFI) level has a marked effect on the success of implantation in ICSI cycles [47]. It is also in line with the recent suggestions that measurement of sperm DNA damage including oxidation level could be used as a predictor of ART outcome [48], [49].

## Materials & Methods

### Animals-Ethics Statement

The present study was approved by the Regional Ethic Committee for Animal Experimentation (CEMEA-Auvergne; Authorization CE2-04) and adhered to the current legislation on animal experimentation in France. Wild type, *snGPx4−/−*, and *snGPx4;GPx5−/−* (DKO) C57bl/6 male mice either 4 or 8 months old were used throughout the study. Mice were housed under controlled environmental conditions (temperature 22°C, 12-h dark period), fed a basal diet (Global-diet, 2016S, Harlan, Gannat, France) *ad libitum*, and given free access to water. For fertility testing, 10-week old C57bl/6 females were used. All male mice were killed by cervical dislocation.

### Genotyping of Mice by PCR

Genomic DNA was extracted from fingers by ethanol precipitation. Genomic DNA was PCR amplified in a final volume of 25 µL in the presence of 2.5 mM MgCl_2_, 400 µM dNTPs, 400 nM of each primer and 1.25U GoTaq Flexi DNA polymerase (Promega) by running 2 independent PCR reactions for each gene as described previously [26]. The primers used in this study are described in the Table 2 and in Figure 1A–B.

### Fertility Measurements and Spermatozoa Samples

*Wt* and *sngpx4;gpx5^−/−^* male mice (five each), aged 3 to 17 months, were mated with one C57bl/6 *wt* synchronized female. At the end of the 7-day reproductive period, males were removed and females were observed to follow eventual pregnancies and deliveries. Time to conceive and number of pups per litter were monitored. For sperm preparations, mice were sacrificed. Epididymides were removed, divided into caput and caudal regions and transferred to a small glass dish containing M2 medium (Sigma-Aldrich, France). To recover the spermatozoa, the caudae epididymides were repeatedly punctured with a needle. After 5 min incubation to allow for sperm dispersal, these preparations were centrifuged at 300 g for 5 min, and pellets were resuspended in phosphate buffered saline (PBS). Sperm counts were determined using a Malassez hemocytometer.

### Cytochemical Tests for Sperm Integrity-Toluidine Blue

DNA compaction was studied by using the modified protocol of Conrad’s group for Toluidine Blue staining [26], where spermatozoa were stained with 1% TB in McIlvain buffer (200 mM Na_2_HPO_4_, 100 mM citric acid, pH 3.5) for 17 min at room temperature. Slides were dehydrated in ethanol and mounted with Cytoseal 60 medium. Three smears per sample were deposited on glass plates and at least 300 spermatozoa per smear were counted. *Shorr staining* – The sperm morphology was analyzed by Shorr staining [fixation in 70% (v/v) aqueous ethanol for 1 min; running water 2 min; Mayer's Hematoxylin (DiaPath) 3 min; running water 3 min; ammoniacal alcohol 10 s; running water 3 min; 70% ethanol 10 s; 95% ethanol 10 s; Shorr stain (Merck) 1 min; 95% ethanol 10 s; 100% ethanol twice for 10 s; Histo-Clear (National Diagnostics) 10 s; mounting with Richard-Allan Scientific® Cytoseal™ 60 medium (Electron Microscopy Sciences, USA)]. Three smears per sample were deposited on glass plates and at least 300 spermatozoa per smear were counted. *Acrosome and nucleus morphology-* Acrosome was labeled using lectin PNA Alexa Fluor® 488 conjugate (Invitrogen) and DNA was stained by Hoechst 33342 (Invitrogen). Air-dried smears of spermatozoa were hydrated in PBS and then incubated for 30 min in PBS, 50 µg/mL PNA-Alexa 488 at 37°C in the dark. After 2 washes, slides were incubated in Hoechst 33342 (1 µg/mL) for 5 min at room temperature, washed 3 times in PBS and mounted with PBS/glycerol (1∶1).

### Cytometry

Spermatozoa of caput or cauda epididymides were diluted with M2 medium to 10^6^ sperms/ml. Flow cytometer evaluation was performed using a Calibur cytometer (Becton Dickinson, Le Pont de Claix, France). For each analysis, 10,000 events were counted using specific probes as described thereafter. Argon laser excitation at 488 nm was coupled with emission measurements at 530/30 (red) and 585/42 (green) band pass, respectively. Non-specific sperm events were gated out. The data were analysed with logiciel CellQuest Pro software (Becton Dickinson, Le Pont de Claix, France). Percentages of living and dead cells were assessed using propidium iodide (PI) (0.01 mg/ml, Sigma). PI was incubated with sperm cells for 8 min at 37°C, then cells were analysed by cytometry on FL3 channel (>650 nm). Sperm total thiol contents were determined using monobromobimane as a probe (mBrB [Thiolyte], Calbiochem, VWR, Fontenay sous Bois, France). A 50 mM solution stock was diluted in 100% acetonitrile. Sperm cells were stained with 1 mM mBrB for 30 min at 25°C then washed twice with PBS. Free thiols were measured by fluorescence on FL1 channel (515–545 nm). To evaluate sperm chromatin condensation, sperm cells were stained with 0.25 mg/ml chromomycin A3 (CMA3, Sigma, France) for 20 min at 25°C. Sperm nuclei decondensation level was measured by cytometry on FL2 channel (564–606 nm).

### Peroxidase Activity Assay

Analyses of peroxidase activity were performed as previously described [38] using hydrogen peroxide (200 µM) as a substrate and glutathione (3 mM)/glutathione reductase (1.4 U). NADPH oxidation was monitored at 340 nm.

### Lipid Peroxidation Assay

Lipid peroxidation (LPO) in the tissues was measured by thiobarbituric acid reacting substance (TBARS) and was expressed in terms of malondialdehyde (MDA) content. Sample aliquots were incubated with 10% trichloroacetic acid and 0.67% thiobarbituric acid. The mixture was heated in a boiling water bath for 30 min, an equal volume of n-butanol was added, and the final mixture was centrifuged; the organic phase was collected for fluorescence measurements. Samples assayed for MDA contained 1 mM butylated hydroxytoluene (BHT) in order to prevent artefactual LPO during the boiling step. The absorbance of samples was determined at 532 nm. Results were expressed as µmol MDA g^–1^ protein.

### Sperm DNA Fragmentation Assay

Assessment of sperm DNA fragmentation was carried out using the staining protocol of the Halomax kit (Chromacell, Spain), a modified Sperm Chromatin Dispersion Assay [28]. Four smears per sample were deposited on glass plates and at least 500 spermatozoa per smear were counted.

### Evaluation of DNA Peroxidative Damages

8-hydroxy-2′-deoxyguanosine detection (8-oxodG) was carried out on spermatozoa from cauda epididymidis. Spermatozoa were resuspended in a decondensing buffer consisting of 2 mM DTT and 0.5% Triton X-100 in PBS 1X and incubated for one hour at room temperature. After centrifugation at 300 g for 5 min at room temperature, spermatozoa were washed in PBS and smeared on a glass plate (30.000 cells/plate). WT and DKO spermatozoa were compared for their reactivity towards the 8-oxodG monoclonal antibody (15A3, Novus biological, Interchim, France). As a positive control of oxidative damage of sperm DNA, a WT spermatozoa aliquot was treated for 2 hours at room temperature by 0.02% H_2_O_2_. Incubations with the primary antibody (dilution 1/500) were conducted overnight at 4°C. Then, after two washes in PBS 1X, Triton X-100 0.1%, the secondary antibody was applied for 1 hour at room temperature (dilution 1/500, anti-mouse IgG polyclonal coupled HRP, P.A.R.I.S Anticorps, France). Signal detection was obtained by the use of the Vector Nova Red substrate kit for peroxidase (Vector Laboratories, AbCys, Paris, France). Two smears per sample were deposited on glass plates and at least 300 spermatozoa per smear were counted.

**Table 2:** Primer sequences used for genotyping of transgenic animals and real time PCR amplifications of selected thiol peroxidases and antioxidant-related genes.

Target	GenBank	5′-3′ primers sequences	Size (bp)	Melting (°C)
**Genotyping**				
*sngpx4*	NC_000076.5	P1/F – TCGGCGGCGCCTTGGCTACCGGCTCP2/R – GGATCCGCCGCGCTGTCTGCAGCGTCCCP3/R – TGAAGAAGTCGTGCTGCTTCATGTGG	119342	60
*gpx5*	NC_000079.5	P4/F – GTGTCTGAGAATCTAGTCCTAGCP5/R – GTGACAGTTTTCTCAGGGGTTGGP6/R – CTGCCTTGTGAAGGTTGACAGG	2631498/278	60
**q-PCR**				
*cyclophilin B*	NM_011149.2	F – GGAGATGGCACAGGAGGAAR – GCCCGTAGTGCTTCAGCTT	76	57 à 62
*gpx1*	NM_008160.5	F – GTCCACCGTGTATGCCTTCTR – CTCCTGGTGTCCGAACTGAT	217	62
*gpx3*	NM_008161.2	F – TCGGAGATACTCCCCAGTCR – AGTTCCAGCGGATGTCATGG	211	58
*gpx4*	NM_008162.2	F – AGTACAGGGGTTTCGTGTGCR – CGGCAGGTCCTTCTCTATCA	410	62
*Catalase*	NM_009804.2	F – GCAGATACCTGTGAACTGTCR – GTAGAATGTCCGCACCTGAG	229	62
*sod3*	NM_011435.3	F – GCTCTCAGAGAACCCCTCTR – GTGCTATGGGGACAGGAAGA	170	58
*prdx1*	NM_011034.4	F – CAACTGCCAAGTGATTGGCGR – TGAGCAATGGTGCGCTTGGG	135	59
*prdx2*	NM_011563.5	F – TGACTTCACGGCCACAGCGGR – CGGAAGTCCTCAGCATGGTC	159	61
*prdx3*	NM_007452.2	F – CAGACATACTGTGGTCTGCCR – AAGTCGTCGAGACTCAGCTC	151	59
*prdx4*	NM_016764.4	F – CAGGACATACTCTTAGAGGCCR – TCACTACCAGGTTTCCAGCC	178	62
*prdx5*	NM_012021.2	F – CGTGCATCGACGTGCTTGGCR – ACCTCCACTGAGGGAATGGC	137	60
*prdx6*	NM_007453.3	F – CACAGAACTTGGCAGAGCTGR – TCGACTGGATCCAACATGCC	219	59
*txn1*	NM_011660.3	F – TGGTGAAGCTGATCGAGAGCR – GGAATACTTGTCACAGAGGG	149	59
*txn2*	NM_019913.5	F – GGTGGTCATGGCCAAAGTGGR – CTTCTAGCTGGTCCTCGTCC	146	61
*txnl1*	NM_016792.4	F – ACTGTGGCATTCAATCAGCCR – AGTTGGCTCACTCCTTTCCG	144	57
*txndc2 (sptrx1)*	NM_001146002.1	F – GGAGCTCCTGAAGAGTCGGR – GGCCTTCTCTTTGGACTGGG	150	62
*txndc3 (sptrx2)*	NM_181591.3	F – CCCTGAAGAGGTAGTGAGGGR – GGTGCAAACCTAACGTGAGGC	207	59
*txndc8 (sptrx3)*	NM_026132.2	F – GTTTGCTCAGGTGGATGTGGR – CTTCGGTCCACTTCTGAGGC	154	62
*gst µ*	NM_010358.5NM_008183.3	F – GAAGTTCAAGCTGGGCCTGGR – GCATGATGAGCTGCATGCGG	194	59
*gst π*	NM_013541.1NM_181796.2	F – AGCTTTCATCGTGGGTGACCR – GGGACGGTTCACATGTTCCG	187	59
*pdia3 (erp57)*	NM_007952.2	F – GGACATTGCAAGAGGCTTGCCCR – TAGGCCCATCATAAGCACCCGC	184	62
*pdia4 (erp72)*	NM_009787.2	F – TGATGGCTCCAGGACCCAGGR – TTGCTGAGCTCCTTGGCAGC	222	61
*pdia5*	NM_028295.1	F – TTTCCAGAAGGCTGCCACCCR – CTCCACGATGTCCTCGGCC	190	62
*pdia6 (txndc7)*	NM_027959.3	F – TGGAAGAAAGCAGCAACGGCR – CATCTACAATGGCTTCTCCC	184	57
*pdia10 (txndc4/erp44)*	NM_029572.2	F – CAGATTGCCCTGTCATAGCCR – TGTCAGTCGGGTCAGGTCCG	159	57
*pdia11 (txndc1)*	NM_028339.1	F – GCTTGTCAGAATCTTCAGCCR – CACATAGCGCCTAAATTCACC	174	60

For each gene studied are indicated the Genbank accession number of the sequences used to design primers, the primers sequences (forward: F; reverse: R), the fragment size and the annealing temperature.

### Microscopy

Observations and counts of Shorr staining, Toluidine blue staining and DNA fragmentation assay were made in transmitted light with the Axioskop (Carl Zeiss, Germany), using magnification of x400. 8-oxodG-positive spermatozoa were counted by Axioplan2 imaging (Carl Zeiss, Germany) in transmitted light (magnification: x630). Observations of fluorescent probes were made by Axioplan2 imaging (Carl Zeiss, Germany) at excitation and emission wavelengths: Alexa 488, BP 475/40 and BP 530/50; Hoechst 33342, BP 365/12 and LP 397, respectively (magnification: x1000).

### Quantitative Reverse Transcriptase Polymerase Chain Reaction

Total RNAs were isolated with the NucleoSpin® RNA II kit (Macherey-Nagel, France). Total RNAs were reverse transcribed by M-MLV Reverse Transcriptase (Promega Corp., France) according to the manufacturer’s instruction. Quantitative real time PCR assays were performed using a RealPlex thermocycler (Eppendorf). Two µL of diluted cDNA template (1/20 caput epididymidis; 1/5 cauda epididymidis) were amplified using MESA GREEN qPCR MasterMix Plus (Eurogentec, France) according to the manufacturer’s instructions. Primer sequences are given in Table 2. A standard curve of amplification efficiency for each set of primers was generated with a serial dilution of plasmids containing DNA of targeted genes. Melting curve analysis was carried out to confirm the specificity of primers. For quantification of transcripts, the relative method was used to calculate mRNA relative level to *Cyclophilin B* standard.

### Statistical Analysis

*Kruskal-Wallis* and *Mann-Whitney* nonparametric tests were performed with GraphPad Prism 5.02 software to determine the significance of differences between samples. *P* values of ≤0.05 were regarded as significant.
