# Correction: Epididymis Response Partly Compensates for Spermatozoa Oxidative Defects in snGPx4 and GPx5 Double Mutant Mice

**DOI:** 10.1371/annotation/a546c716-7c58-4b69-a19f-62629cb72693

**Published:** 2012-09-21

**Authors:** Anaïs Noblanc, Manon Peltier, Christelle Damon-Soubeyrand, Nicolas Kerckhove, Eléonore Chabory, Patrick Vernet, Fabrice Saez, Rémi Cadet, Laurent Janny, Hanae Pons-Rejraji, Marcus Conrad, Joël R. Drevet, Ayhan Kocer

The fourth author's name was spelled incorrectly. The correct name is: Nicolas Kerckhove.

The correct citation is: Noblanc A, Peltier M, Damon-Soubeyrand C, Kerckhove N, Chabory E, et al. (2012) Epididymis Response Partly Compensates for Spermatozoa Oxidative Defects in snGPx4 and GPx5 Double Mutant Mice. PLoS ONE 7(6): e38565. doi:10.1371/journal.pone.0038565 

